# Research hotspots and frontiers of cluster headaches: a bibliometric analysis

**DOI:** 10.3389/fneur.2024.1395770

**Published:** 2024-04-25

**Authors:** Qiangjian Mao, Shiqi Xu, Yuqing Wang, Desheng Wu, Guomin Huang, Ziru Li, Xiaoming Zhang, Zhenhai Chi

**Affiliations:** ^1^Department of Acupuncture and Moxibustion, Affiliated Hospital of Jiangxi University of Chinese Medicine, Nanchang, Jiangxi, China; ^2^Acupuncture and Moxibustion Massage College, Jiangxi University of Chinese Medicine, Nanchang, Jiangxi, China

**Keywords:** cluster headache, bibliometrics, visualization, hotspots, frontiers

## Abstract

**Background:**

Extensive research on cluster headaches (CHs) has been conducted worldwide; however, there is currently no bibliometric research on CHs. Therefore, this study aimed to analyze the current research hotspots and frontiers of CHs over the past decade.

**Methods:**

Raw data on CHs was obtained from the Web of Science Core Collection database from 2014 to 2023. CiteSpace V6.2 R7 (64 bit) and Microsoft Excel were used to assess the annual publication volume, authors, countries, and references. VOSviewer 1.6.19 software was used to assess the institutions, cited authors, and keywords, and co-occurrence and clustering functions were applied to draw a visual knowledge map.

**Results:**

In the past decade, the overall annual publication volume of articles related to CHs has increased year by year, showing promising development prospects. The total 1909 articles contained six types of literature, among which the proportion of original research articles was the highest (1,270 articles, 66.53%), published in 201 journals. *Cephalalgia* (439 articles, 23.00%) had the highest publication volume, and the *Lancet* was the journal with the highest impact factor (IF = 168.9). Furthermore, the United States of America was the country with the most published papers (584 articles, 30.60%), University of London was the research institution with the most published papers (142 articles, 7.44%), and Goodsby, Peter J was found to be the most prolific author (38 articles, 1.99%).

**Conclusion:**

This study may provide some direction for subsequent researcher on CHs. The hotspots and frontiers of future research on CHs are suggested as follows: in basic medicine, more attention should be paid to pathophysiology, especially on increasing research on the pathogenesis mediated by CGRP; in clinical medicine, more attention should be paid to the design of evidence-based medicine methodology, especially the strict design, including double-blind, questionnaire, and follow-up, in randomized controlled trials, using high-quality articles for meta-analyses, and recommending high-level evidence; therapeutic techniques need to be further explored, suggesting the implementation of transcranial magnetic stimulation of the cortex, and stimulation of the sphinopalatine ganglia and occipital nerve to achieve peripheral neuromodulation. Furthermore, chronic migraine and insomnia are inextricably linked to CHs.

## 1 Introduction

A cluster headache (CH) is a severe primary headache and the most common type of primary headache disease (1). The characteristics of a CH are severe or extremely severe pain in the unilateral orbit, supraorbital, and/or temporal regions, accompanied by autonomic nervous symptoms, such as conjunctival congestion, tearing, eyelid drooping, or restlessness, restlessness, or both, lasting 15–180 min. It usually occurs once every other day or up to eight times per day (2, 3). Epidemiological surveys conducted in the United States of America (USA) and Europe have reported that the prevalence of CHs in the general population is approximately 0.1% (4), and a lifetime prevalence rate of 0.12% (5). A large-scale survey found that CHs impose a significant personal burden on patients, with nearly 20% of patients losing their jobs and an additional 8% being unemployed or disabled (6). In addition, female patients with CHs are more prone to disability than males, with over 50% of women developing suicidal thoughts and 2% attempting suicide (7). Although the incidence of CHs is low, a single severe or recurrent headache attack can seriously affect the quality of life and work ability of patients. Therefore, raising public awareness of CHs is of great significance (8).

The pathological and physiological mechanisms of CHs are complex, with most research focused on the trigeminal neurovascular system, trigeminal autonomic reflex, and hypothalamic activation (9). Genetic factors have also been reported to be involved the pathological and physiological processes of CHs (10). The main treatment for this disease is drug staging (11). Triptans and high-flow oxygen inhalation through masks are used for acute phase treatment (12), steroids are used for transitional phase treatment, and verapamil and glucocorticoids are commonly used as preventive treatments (13, 14). In clinical practice, drug treatment of CHs only temporarily relieves symptoms, leaving patients prone to recurrent attacks (15). Therefore, researchers should pay more attention to this disease, increase research investment, and actively seek alternative therapies (16).

Bibliometrics is the study of various external characteristics of the literature (17, 18), which is performed through mathematical and statistical quantitative analysis methods, objectively reflecting the state of research in the field (19, 20). This avoids the subjective evaluation of the researcher (21); therefore, bibliometrics has now become an important tool for global analyses and investigations in various fields (21).

To our knowledge, there is currently no bibliometric analysis on CHs. Therefore, this study aimed to analyze the literature on CHs in the past decade with the goal to comprehensively grasp the research status and academic hotspots and frontiers of CHs worldwide.

## 2 Materials and methods

### 2.1 Source of literature

**Figure 1:**
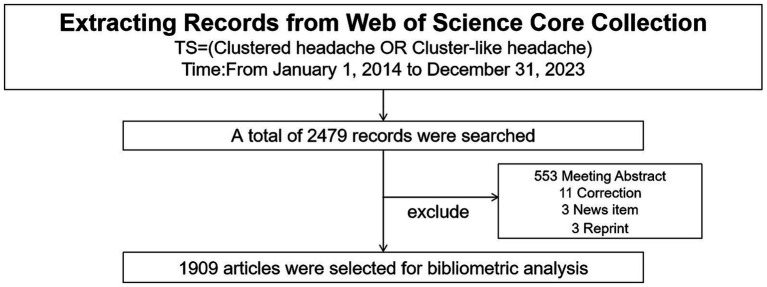
Flow chart of the included study.

We used the Web of Science Core Collection (WOSCC) database to conduct the search using the search terms “cluster headache” or “cluster-like headache” from January 1, 2014, to December 31, 2023. The search was completed independently by two authors, Yuqing Wang and Desheng Wu; Shiqi Xu was responsible for unifying and resolving any disputes as they arose. We retrieved a total of 2,479 articles, which were imported into CiteSpace software. No duplicate articles were detected. To ensure the reliability of research conclusions, we included as many articles as possible, with no restrictions on the type and language of the articles; however, short passages or incomplete articles were not included in the analysis. Therefore, we subsequently removed 553 meeting abstracts, 11 corrections, three news items, and three reprints. Data not included in the analysis were deleted using CiteSpace software after confirmation by Shiqi Xu. In the end, 1909 articles related to CHs were used for the bibliometric analysis (Figure 1). The Web of Science database was obtained from the library of Jiangxi University of Chinese Medicine.

### 2.2 Analysis tools

CiteSpace is an analytical software developed by Dr. Chen Chaomei from Drexel University in the USA (22). It is based on mathematics, graphics, statistics, and informatics, and uses econometric methods to analyze specific research field literature and draw corresponding visual knowledge graphs to form a deep analysis of the field (23, 24). VOSviewer is a free software jointly developed by Dr. Van Eck and Dr. Waltman from Erasmus University in the Netherlands (25). It can be used to perform collaborative network analysis, co-occurrence analysis, citation analysis, literature coupling analysis, and co-citation analysis on the literature (26). Its advantage lies in its ability to handle a large amount of data, and the co-occurrence network operates normally when there are many nodes (27). The generated graph is relatively clear and has strong readability (28). Both software tools visualize the literature and present the history, patterns, and structural relationships of knowledge evolution in a certain scientific discipline or research field (29), helping researchers to understand and master the development direction and trace the forefront of a certain scientific field (30).

CiteSpace V6.2.R7 (64-bit) and Microsoft Excel were used to draw distribution maps of annual publication volume, authors, countries, and references. VOSviewer 1.6.19 software was used to draw distribution maps of institutions, cited authors, and keywords.

## 3 Results and discussion

### 3.1 Analysis of the annual volume of publications

**Figure 2:**
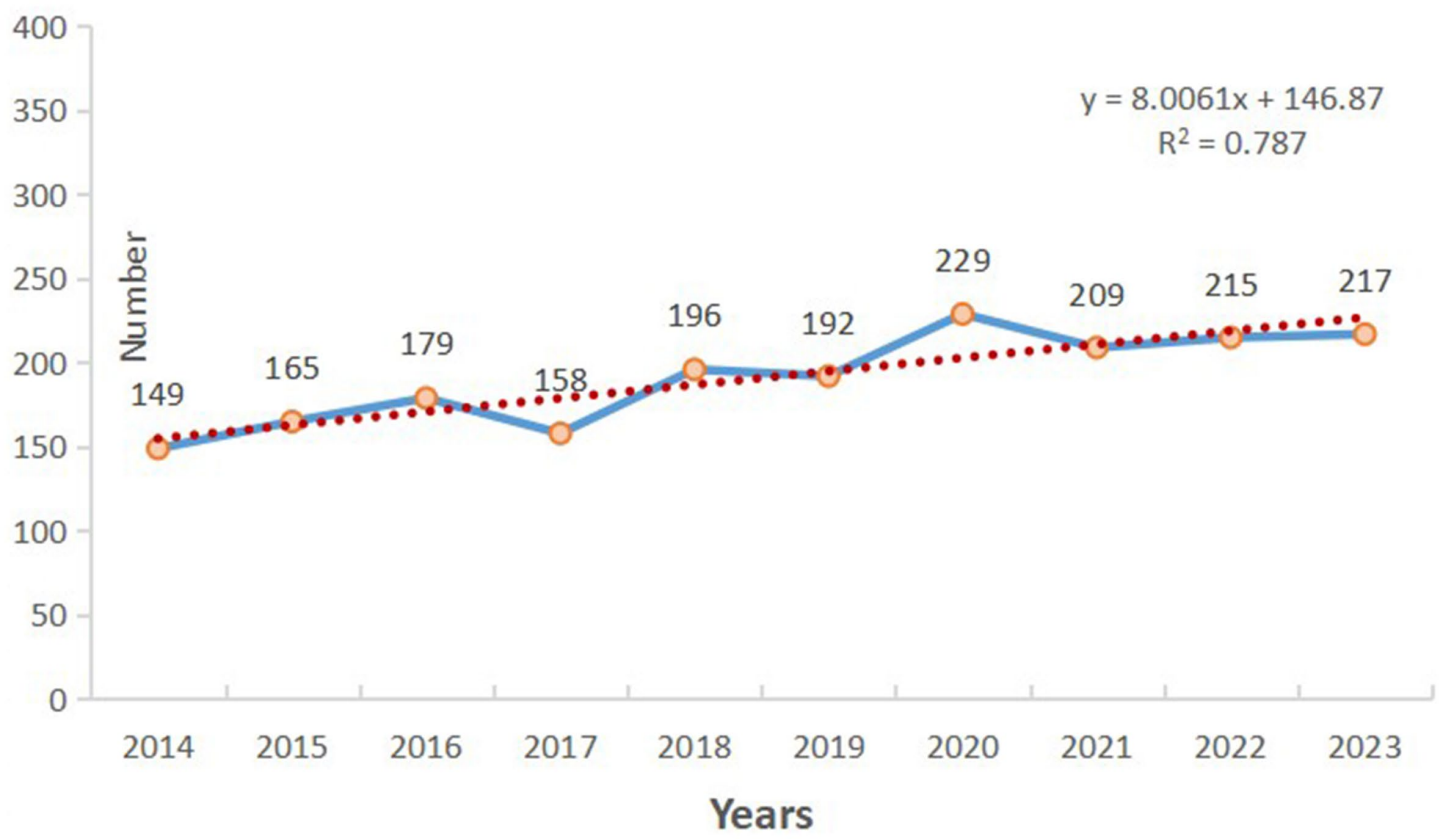
The annual number of publications related to cluster headaches (CHs) from 2014 to 2023.

Microsoft Excel was used to draw an annual publication quantity distribution map, which can be used to understand the changes and developments in this research field to a certain extent (Figure 2). In the past decade, the number of articles related to CHs has fluctuated slightly, but overall, it has shown a continuous upward trend. We have demonstrated high reliability of the trend line by calculating the slope (y = 8.0061x + 146.87; R^2^ = 0.787). It is worth noting that the number of articles published reached a historical peak in 2020 (229 articles, 12.00%). The research results indicate that CHs have attracted the attention of researchers and have good development prospects.

### 3.2 Analysis of journals and cited journals

The 1909 articles were categorized into six types of publication. The most common type of publication was original research articles (1,270 articles, 66.53%), followed by reviews (457 articles, 23.94%), editorial material (72 articles, 3.77%), letters (46 articles, 2.40%), early access (40 articles, 2.10%), and proceeding papers (24 articles, 1.26%) (Table 1). The 1909 articles were published in 201 journals, with the highest number of articles published in *Cephalalgia* (439 articles, 23.00%), followed by *Journal of Headache and Pain* (262 articles, 13.72%), *Headache* (250 articles, 13.10%), *European Journal of Neurology* (69 articles, 3.61%), and *Neurology* (67 articles, 3.51%) (Table 2 shows the top 10 journals). Based on the latest 2023 Journal Citation Report from the American Institute of Scientific Information, we concluded that among these journals, the *Lancet* was the journal with the highest impact factor (IF = 168.9).

**Table 1:** Literature types related to cluster headaches (CHs).

Rank	Type	Counts (%)	Rank	Type	Counts (%)
1	Article	1,270 (66.53)	4	Letter	46 (2.40)
2	Review	457 (23.94)	5	Early Access	40 (2.10)
3	Editorial Material	72 (3.77)	6	Proceedings Paper	24 (1.26)

**Table 2:** Top 10 journals and publications related to cluster headaches (CHs).

Rank	Publications	Journal	IF (2023)	Rank	Publications	Journal	IF (2023)
1	439	Cephalalgia	4.9	6	57	Neurological Science	3.3
2	262	Journal of Headache and Pain	7.4	7	50	Current Pain and Headache Reports	3.7
3	250	Headache	5.0	8	37	Frontiers In Neurology	3.4
4	69	European Journal of Neurology	5.1	9	23	Neuromodulation	2.8
5	67	Neurology	9.9	10	21	PLoS One	3.7

**Figure 3:**
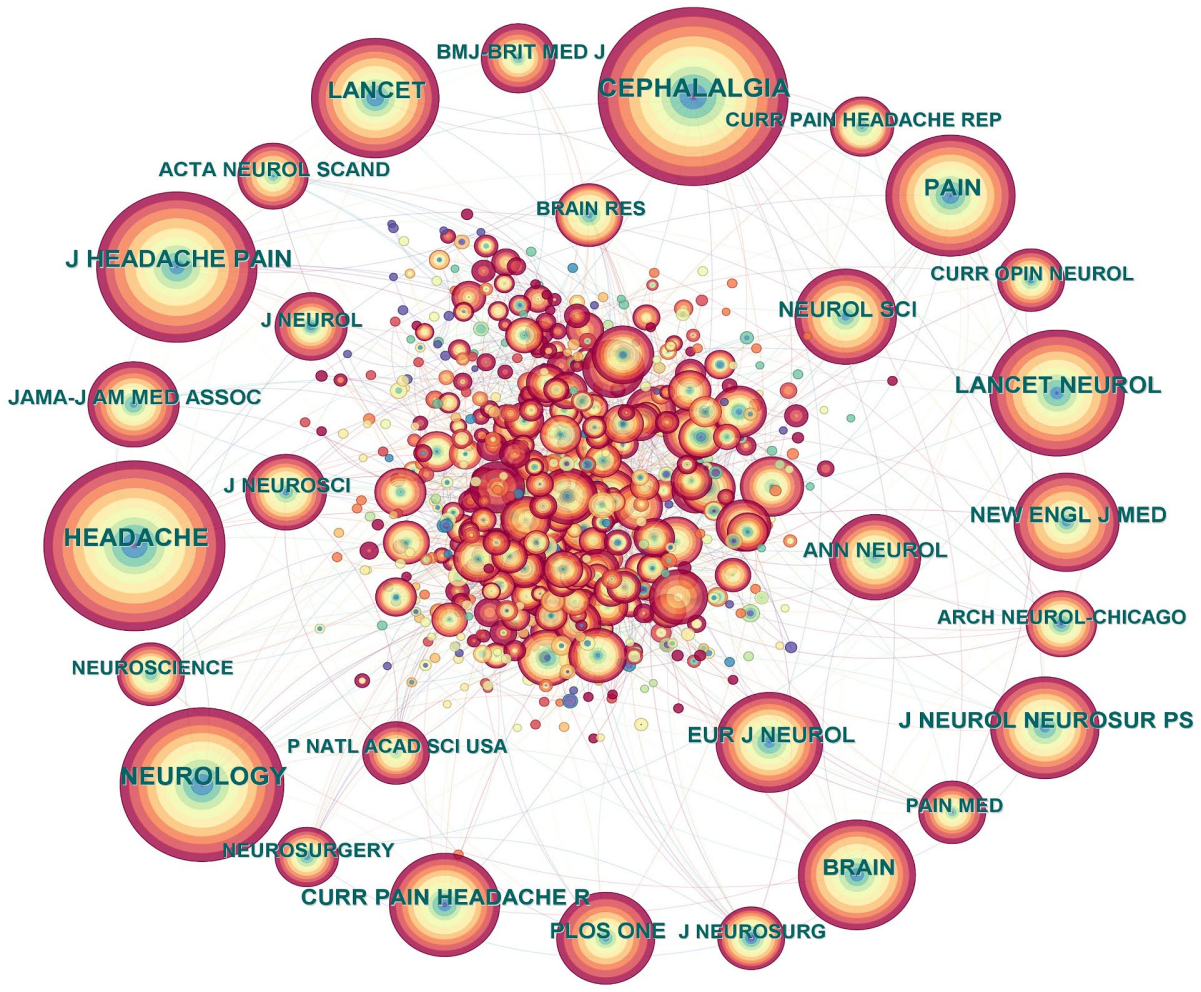
Cited journal map related to cluster headaches (CHs) from 2014 to 2023.

Combining co-citation and centrality, CiteSpace generated a network map of cited journals (Figure 3; Table 3). Through the figures and tables, we can easily see that *Cephalalgia* had the highest frequency of citations and *Annals of Internal Medicine* has the highest centrality. These two journals have significant academic influence and high professional recognition in the field of CH research, providing professional opinions for users to make decisions.

**Table 3:** Top 10 cited journals and centrality related to cluster headaches (CHs).

Rank	Cited Journal	Frequency	Rank	Cited Journal	Centrality
1	Cephalalgia	1,459	1	Annals of Internal Medicine	0.07
2	Headache	1,338	2	Brain Research Bulletin	0.07
3	Neurology	1,112	3	Neuropharmacology	0.05
4	Journal of Headache and Pain	1,080	4	Anesthesia & Analgesia	0.05
5	Lancet Neurol	779	5	Pharmacology & Therapeutics	0.05
6	Pain	712	6	Experimental Neurology	0.05
7	Lancet	710	7	Nature Neuronscience	0.04
8	Brain	602	8	European Journal of Neuroscience	0.04
9	Journal of Neurology Neurosurgery and Psychiatry	536	9	Journal of Clinical Investigation	0.04
10	Current Pain and Headache Reports	525	10	Annals of Emergency Medicine	0.04

### 3.3 Analysis of countries and institutions

**Figure 4:**
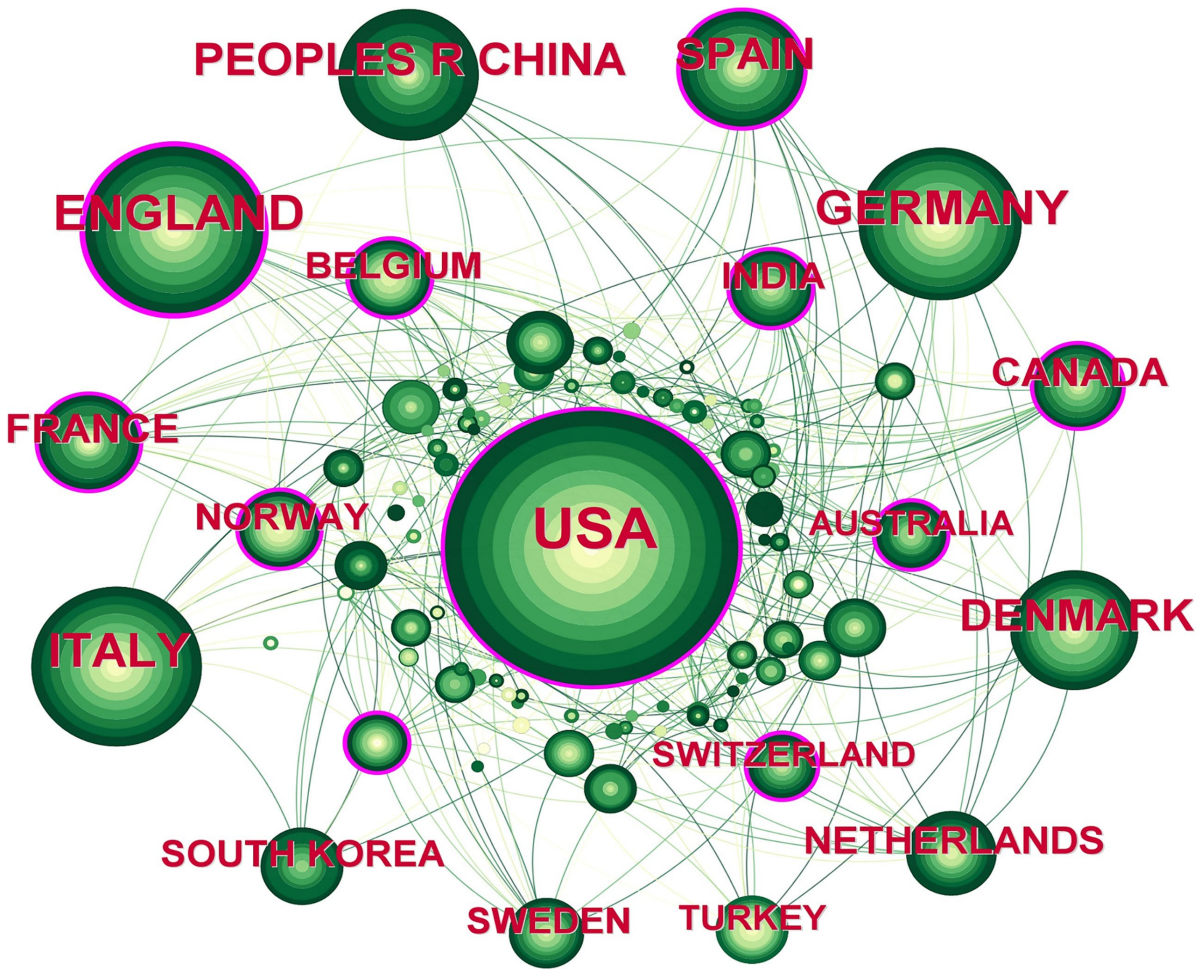
Map of countries researching cluster headaches (CHs) from 2014 to 2023.

The analysis of the countries gives an idea of the global distribution of a research direction. A distribution map of the country cooperative relationship network was generated using CiteSpace, consisting of 100 nodes and 364 connecting lines, representing 1909 articles from 100 countries (Figure 4). The country with the highest number of articles published was the USA (584 articles, 30.60%), followed by England (233 articles, 12.21%), Italy (211 articles, 11.05%), Germany (197 articles, 10.32%), and the People’s Republic of China (147 articles, 7.70%). The highest centrality was found in England (0.36), followed by the USA (0.15), Australia (0.14), and Spain (0.12). The top 10 countries in terms of publication quantity and centrality are shown in Table 4. In summary, extensive research has been conducted in the USA and England on CHs, with a solid research foundation, making significant contributions to the development of research on CHs.

**Table 4:** Top 10 publications and centrality of countries related to cluster headaches (CHs).

Rank	Publications	Countries	Rank	Centrality	Countries
1	584	USA	1	0.36	England
2	233	England	2	0.15	USA
3	211	Italy	3	0.14	Canada
4	197	Germany	4	0.14	Australia
5	147	People’s Republic of China	5	0.12	Spain
6	128	Denmark	6	0.12	Belgium
7	120	Spain	7	0.12	Switzerland
8	85	France	8	0.11	Norway
9	68	Canada	9	0.11	India
10	66	Netherlands	10	0.10	France

**Figure 5:**
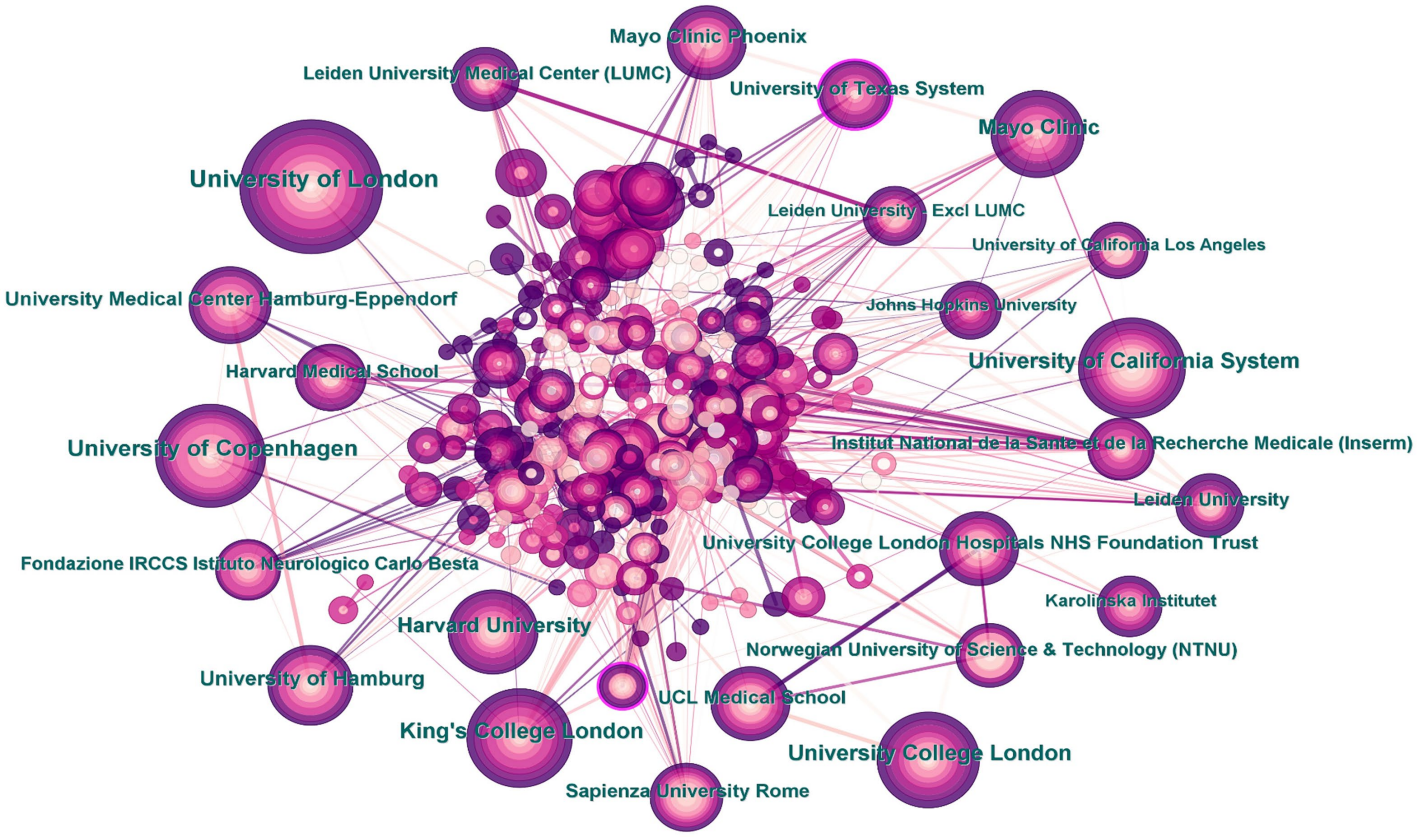
Map of institutions researching cluster headaches (CHs) from 2014 to 2023.

Research institutions are important places for knowledge output. Drawing a distribution map of research institutions can help us to understand the distribution of the main research forces in the research field. The 1909 articles were published by 348 research institutions (Figure 5). The institution with the highest number of published articles was University of London (142 articles, 7.44%), followed by the University of Copenhagen (95 articles, 4.98%), University of California System (83 articles, 4.35%), and University College London and King’s College London (79 articles, 4.14%). The highest centrality was found at the University of Texas System and Istanbul University (0.12), followed by University of Munich and David Geffen School of Medicine at UCLA (0.09), and Johns Hopkins University (0.08). The top ten institutions in terms of article publication volume and centrality are shown in Table 5. The analysis showed that research institutions for CHs are mainly concentrated in comprehensive universities, such as University of London and Harvard University; while research institutes or centers for specific disciplines have a low participation, such as the Max Planck Florida Institute for Neuroscience and Montefior Headache Center. There is more domestic exchange among research institutions and less cross-national exchange.

**Table 5:** Top 10 publications and centrality of institutions related to cluster headaches (CHs).

Rank	Publications	Institutions	Rank	Centrality	Institutions
1	142	University of London	1	0.12	University of Texas System
2	95	University of Copenhagen	2	0.12	Istanbul University
3	83	University of California System	3	0.09	University of Munich
4	79	University College London	4	0.09	David Geffen School of Medicine at UCLA
5	79	King’s College London	5	0.08	Johns Hopkins University
6	69	Mayo Clinic	6	0.08	Assistance Publique Hopitaux Paris (APHP)
7	68	Harvard University	7	0.08	Korea University Medicine (KU Medicine)
8	55	University of Hamburg	8	0.08	CHU Lille
9	53	University Medical Center Hamburg-Eppendorf	9	0.07	King’s College London
10	47	Mayo Clinic Phoenix	10	0.07	Mayo Clinic Phoenix

### 3.4 Analysis of authors and cited authors

**Figure 6:**
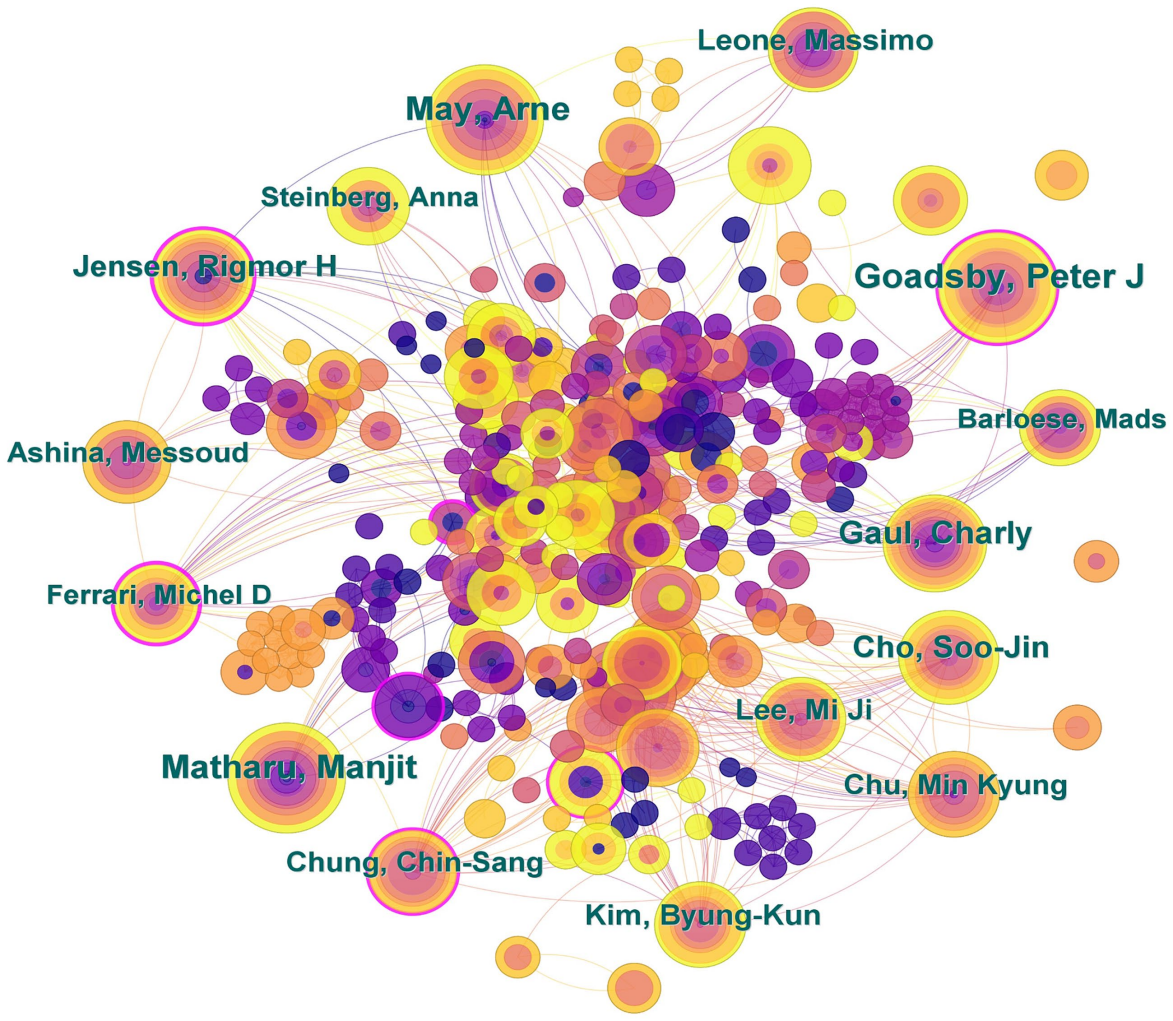
Map of authors related to research on cluster headaches (CHs) from 2014 to 2023.

We put all participating authors in each article into CiteSpace software for analysis and drew a collaborative network map of the authors. This not only gives an idea of the authors’ contribution to the field they are working in, but also allows an analysis of the degree of collaboration between authors (Figure 6). The author with the most publications in studies related to CHs is Goadsby, Peter J (38 articles), followed by Matharu, Manjit and May, Arne (34 articles), Gaul, Charly (28 articles), and Cho, Soo-Jin (26 articles). The top 10 articles are shown in Table 6. A comprehensive analysis showed that Goadsby, Peter J is the most prominent contributor to the field of CH research. The authors have regular partners, and there is a lack of cooperation with other team authors. If authors in different directions can strengthen their connections, CH research will make more breakthroughs.

**Table 6:** Top 10 prolific authors related to cluster headaches (CHs).

Rank	Publications	Author	Rank	Publications	Author
1	38	Goadsby, Peter J	6	23	Jensen, Rigmor H
2	34	Matharu, Manjit	7	21	Kim, Byung-Kun
3	34	May, Arne	8	21	Lee, Mi Ji
4	28	Gaul, Charly	9	21	Leone, Massimo
5	26	Cho, Soo-Jin	10	20	Chu, Min Kyung

**Figure 7:**
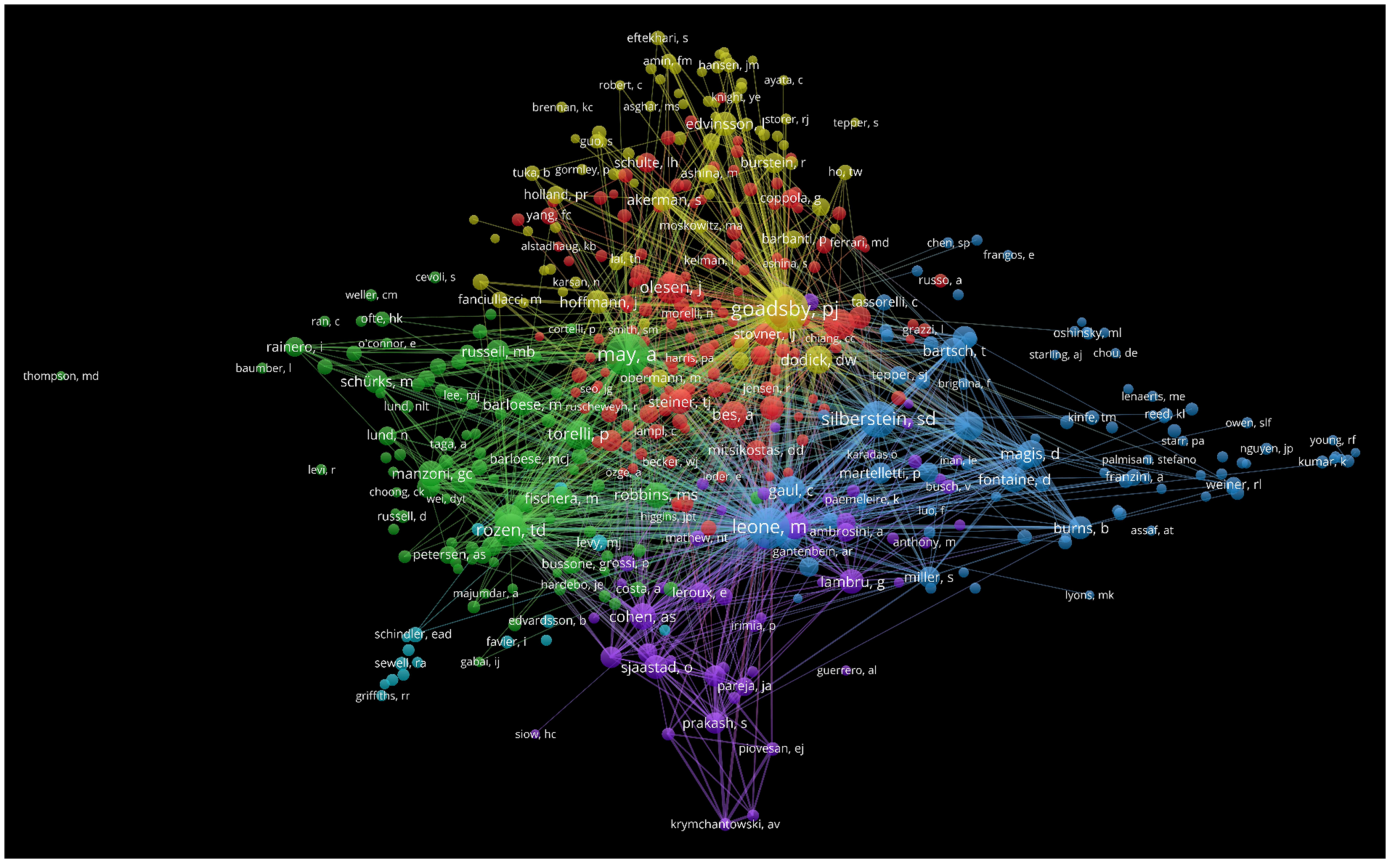
Map of cited authors related to research on cluster headaches (CHs) from 2014 to 2023.

A co-cited network map of authors helps to gain a clear understanding of the core authors in the field of CHs (Figure 7). The most cited authors are Goadsby, PJ (548 articles, 27.15%), followed by May, A (529 articles, 22.25%), Silerstein, SD (476 articles, 20.30%), Leone, M (384 articles, 19.45%), and Olesen, J (361 articles, 15.55%). Busson, G has the highest centrality (0.09), followed by Saper, JR (0.08), Wilbrink, LA (0.07), and Baroese, M and Rapport, AM (0.06). Table 7 lists the top 10 authors in terms of citation frequency and centrality. In summary, Goadsby, PJ and Saper, JR have conducted extensive research on CHs, both showing good research activity and outstanding contributions.

**Table 7:** Top 10 frequency and centrality of cited authors related to cluster headaches (CHs).

Rank	Frequency	Author	Rank	Centrality	Author
1	548	Goadsby, PJ	1	0.09	Bussone, G
2	529	May, A	2	0.08	Saper, JR
3	476	Silerstein, SD	3	0.07	Wilbrink, LA
4	384	Leone, M	4	0.06	Barloese, M
5	361	Olesen, J	5	0.06	Rapoport, AM
6	355	Unknown	6	0.06	Popeney, CA
7	325	Torelli, P	7	0.05	Bigal, ME
8	304	Rozen, TD	8	0.05	Nesbitt, AD
9	302	Bes, A	9	0.05	Cittadini, E
10	265	Lipton, RB	10	0.05	Vanvliet, JA

### 3.5 Analysis of cited references

**Figure 8:**
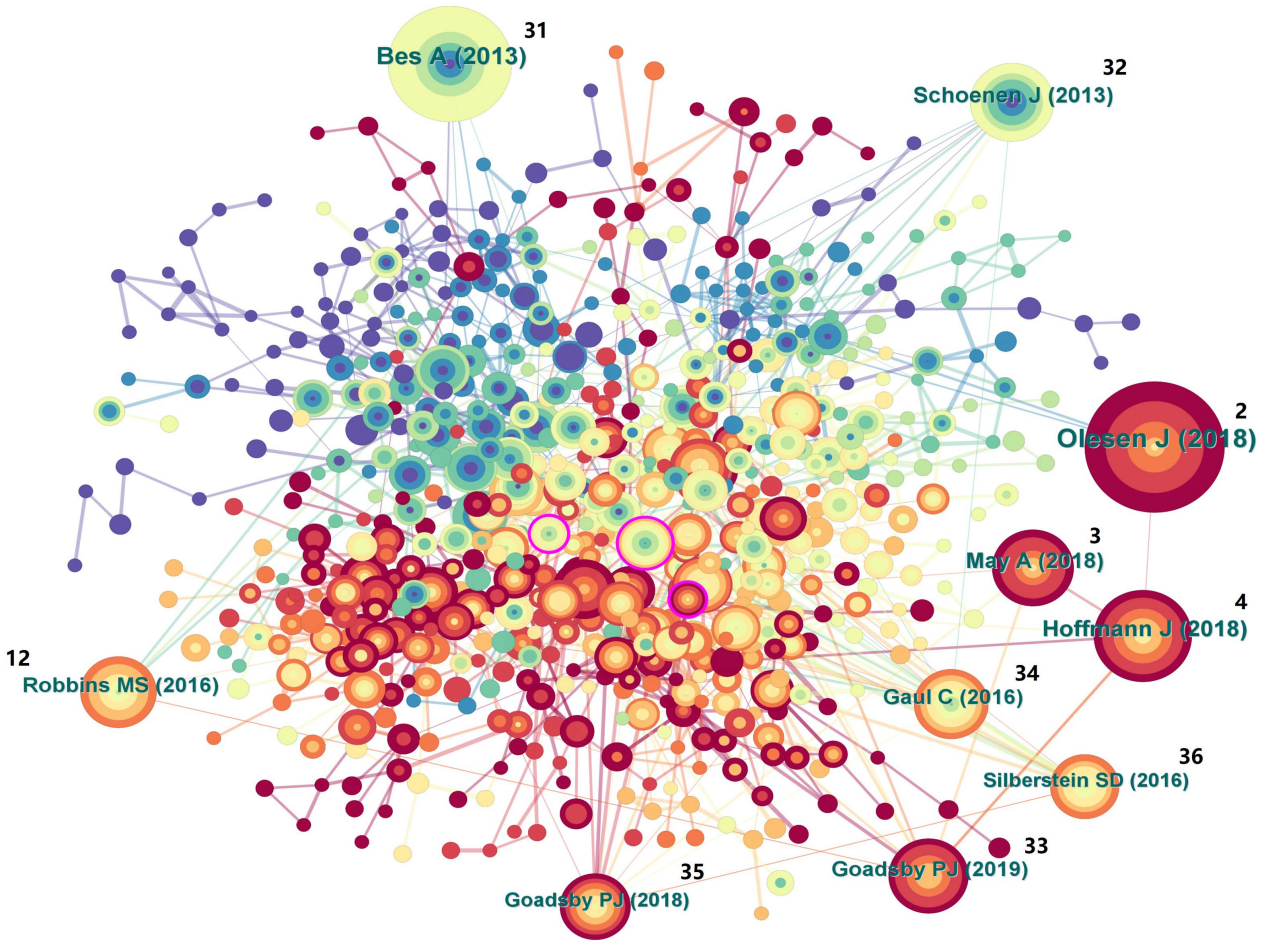
Map of cited references related to cluster headaches (CHs) from 2014 to 2023.

The co-occurrence chart of references can be used to present the quality of literature related to CHs, which is beneficial for researchers to quickly grasp the hot topics in the research field [Figure 8, (2, 31–36) references are labeled in the figure]. The frequency ranking of the top 10 references is shown in Table 8, indicating that these references have a significant impact in the field of CHs. Centrality is the calculation of the likelihood of the shortest path passing through nodes in a network. High centrality presents a purple halo, which helps us quickly identify the most valuable nodes in the network. Table 9 lists the top 10 highly central references, indicating that these references have high quality and a good response in the field of CHs.

**Table 8:** Top 10 frequency of cited references related to cluster headaches (CHs).

Rank	Frequency	References	Author and publication year
1	262	Cephalalgia, V38, P1. DOI: 10.1177/0333102417738202 (2)	Olesen J, 2018
2	199	Cephalalgia, V33, P629. DOI: 10.1177/0333102413485658 (31)	Bes A, 2013
3	128	Lancet Neurol, V17, P75. DOI: 10.1016/S1474-4422(17)30405-2 (4)	Hoffmann J, 2018
4	94	Cephalalgia, V33, P816. DOI: 10.1177/0333102412473667 (32)	Schoenen J, 2013
5	91	Nat Rev Dis Primers, V4, P0. DOI: 10.1038/nrdp.2018.6 (3)	May A, 2018
6	89	New Engl J Med, V381, P132. DOI: 10.1056/NEJMoa1813440 (33)	Goadsby PJ, 2019
7	83	Headache, V56, P1093. DOI: 10.1111/head.12866 (12)	Robbins MS, 2016
8	75	CEPHALALGIA, V36, P534. DOI: 10.1177/0333102415607070 (34)	Gaul C, 2016
9	68	Cephalalgia, V38, P959. DOI: 10.1177/0333102417744362 (35)	Goadsby PJ, 2018
10	66	Headache, V56, P1317. DOI: 10.1111/head.12896 (36)	Silberstein SD, 2016

**Table 9:** Top 10 centrality of cited references related to cluster headaches (CHs).

Rank	Centrality	References	Author and publication year
1	0.14	J Headache Pain, V19, P0. DOI: 10.1186/s10194-018-0874-y (37)	Lampl C, 2018
2	0.10	Cephalalgia, V36, P534. DOI: 10.1177/0333102415607070 (34)	Gaul C, 2016
3	0.10	Neurology, V84, P1249. DOI: 10.1212/WNL.0000000000001394 (38)	Nesbitt AD, 2015
4	0.08	Eur J Neurol, V24, P381. DOI: 10.1111/ene.13215 (39)	Miller S, 2017
5	0.07	J Headache Pain, V17, P0. DOI: 10.1186/s10194-016-0660-7 (40)	Tuka B, 2016
6	0.06	Headache, V52, P99. DOI: 10.1111/j.1526-4610.2011.02028.x (6)	Rozen TD, 2012
7	0.06	Cephalalgia, V32, P1165. DOI: 10.1177/0333102412462642 (41)	Silberstein SD, 2012
8	0.06	New Engl J Med, V381, P132. DOI: 10.1056/NEJMoa1813440 (33)	Goadsby PJ, 2019
9	0.05	Cephalalgia, V33, P816. DOI: 10.1177/0333102412473667 (32)	Schoenen J, 2013
10	0.05	Headache, V56, P1093. DOI: 10.1111/head.12866 (12)	Robbins MS, 2016

**Figure 9:**
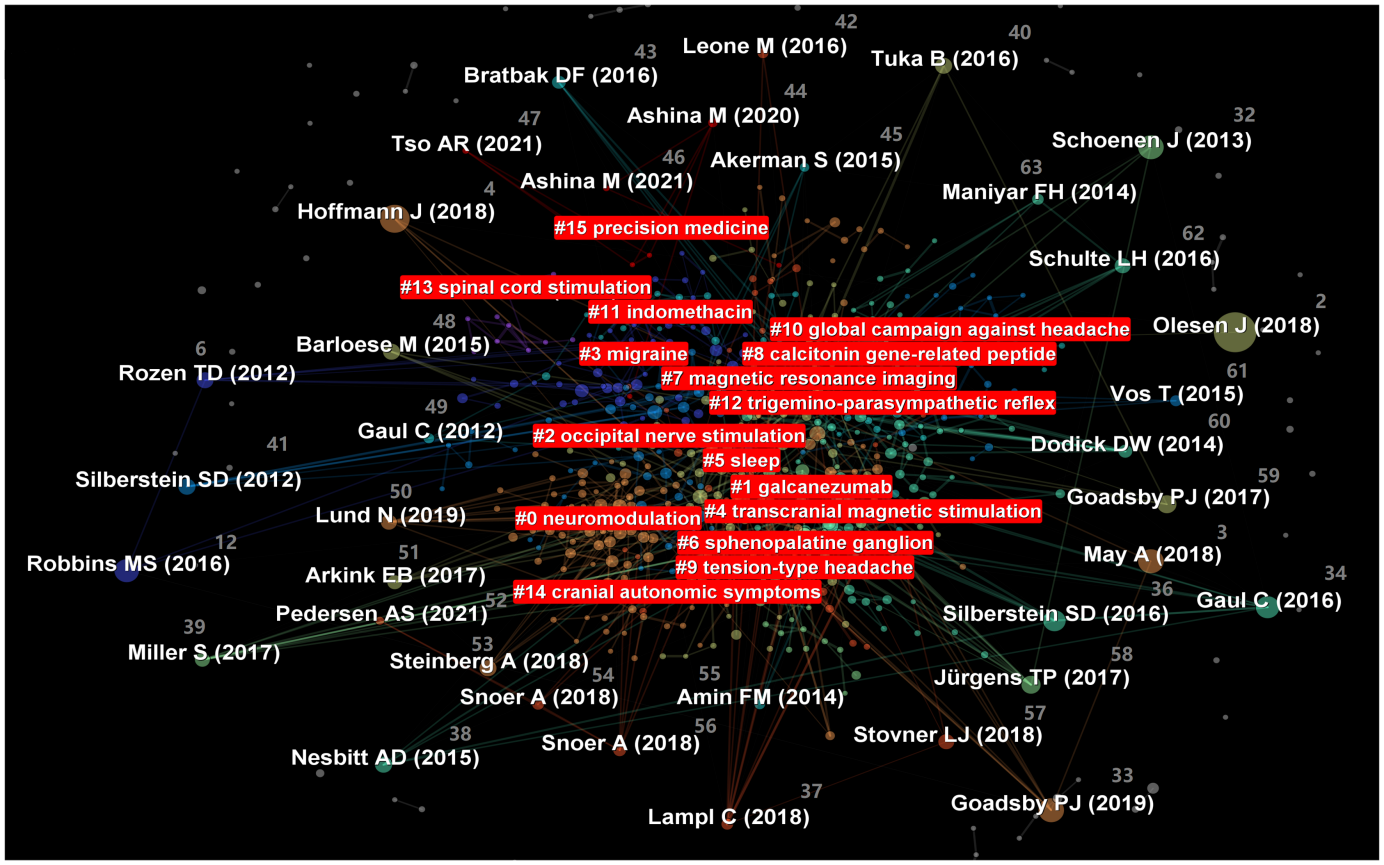
Cluster analysis map of co-citation references related to cluster headaches (CHs) from 2014 to 2023.

To grasp the research progress and dynamics of a certain research field, CiteSpace uses the log-likelihood ratio (LLR) algorithm to generate clustering labels for the network distribution map based on common relationships among references [Figure 9, (37–63) references are labeled in the figure]. In this map, a total of 16 clusters were formed, among which “transcranial magnetic stimulation” and “sphinopalatine ganglia” were two important clustering results. These indicate that transcranial magnetic stimulation and sphinopalatine ganglion stimulation are two particularly important therapeutic techniques for CHs. The cluster modulus, Q = 0.7181 > 0.3, indicated good cluster structure. The average contour value, S = 0.8862 > 0.7, indicates high clustering feasibility. Based on the above analysis, research related to CH is deemed to have high credibility.

Transcranial magnetic stimulation is considered an effective non-pharmacological treatment for CHs. The analgesic effect is suggested to be achieved through a decrease in cortical excitability, the release of beta endorphins, changes in glutamine/glutamate levels, and effects on the hypothalamus (64). A clinical trial has confirmed that researchers have developed a small stimulator to stimulate the sphenopalatine ganglion, which has been proven to be safe and effective in acute and preventive treatments for CHs, with the effect maintained in the long term (65).

### 3.6 Analysis of keywords

**Figure 10:**
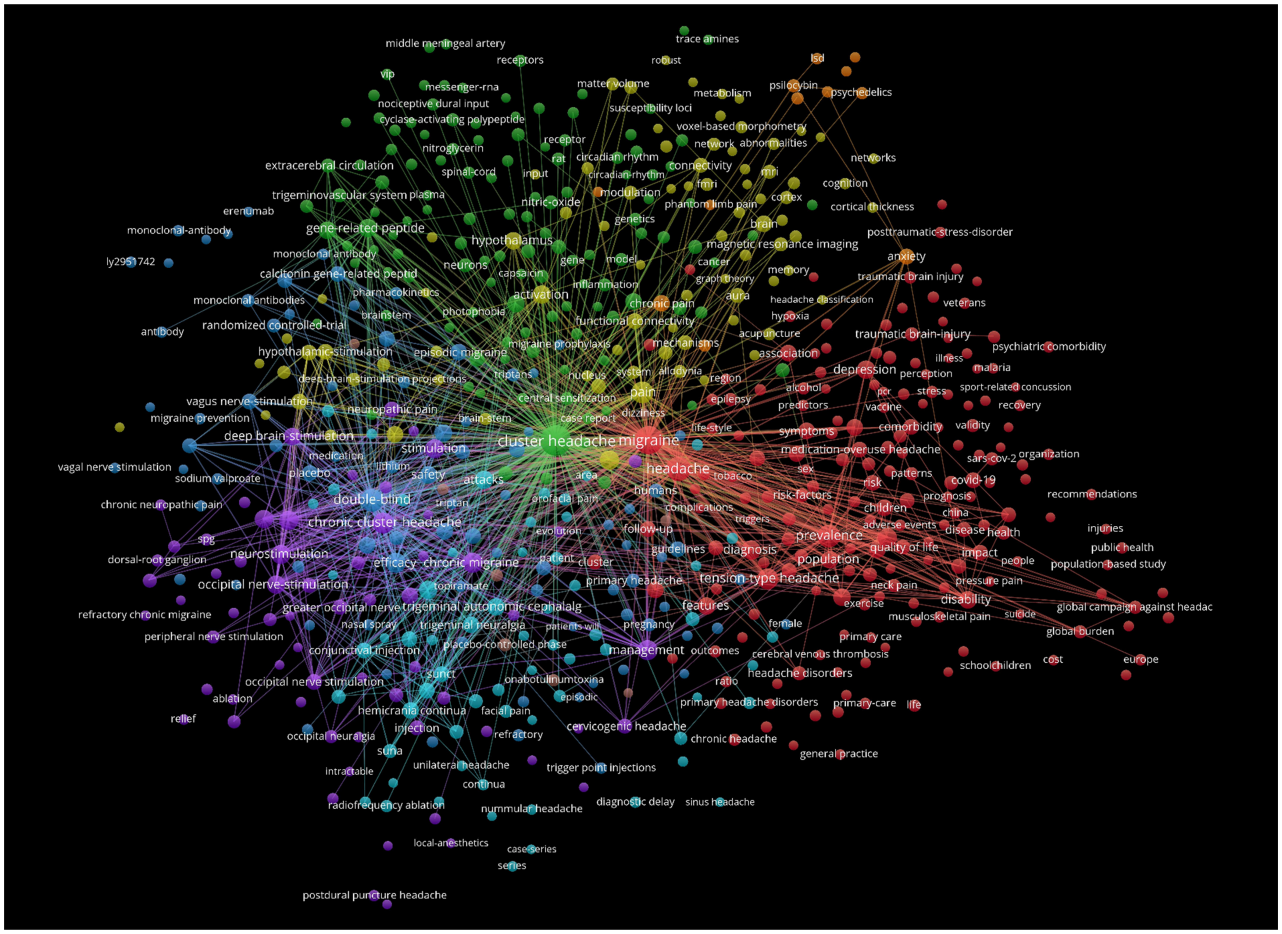
Map of keywords occurrence related to cluster headaches (CHs) from 2014 to 2023.

**Figure 11:**
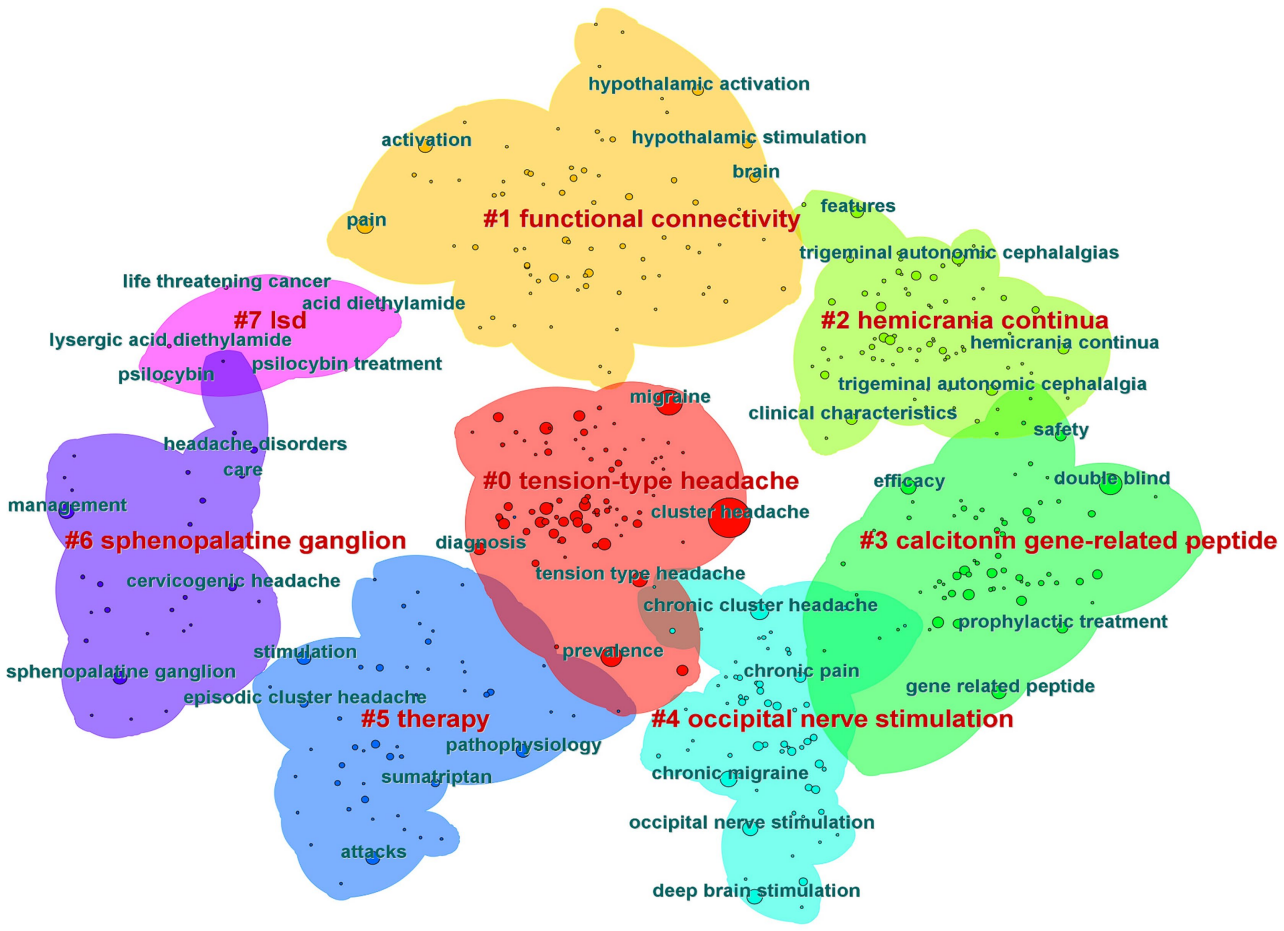
Cluster analysis map of co-citation keywords related to cluster headaches (CHs) from 2014 to 2023.

Keywords are an important component of an article. The co-occurrence analysis of keywords is beneficial for understanding the core viewpoints and hot topics in the field of CH research (Figure 10). We found that “cluster headache,” “migraine,” “double blind,” “prevalence,” and “chronic cluster headache” were the most popular keywords (Table 10). Keyword clustering can, to some extent, be used to summarize the main research clusters related to CHs. In this study, clustering analysis was performed on the obtained keywords using clustering algorithms. A total of eight clusters were formed (Figure 11), among which “calcitonin gene-related peptide” and “occipital nerve stimulation” were two important clustering results. These indicate that research hotspots may be related to pathogenesis and therapeutic techniques of CHs. The cluster modulus, Q = 0.4793 > 0.3, indicated a significant clustering structure. The average contour value, S = 0.7905 > 0.7, indicated reasonable clustering. Overall, research on CHs shows strong readability.

**Table 10:** Top 10 frequency and centrality of keywords related to cluster headaches (CHs).

Rank	Keyword	Frequency	Rank	Keyword	Centrality
1	Cluster headache	856	1	Brain	0.08
2	Migraine	331	2	Episodic cluster headache	0.07
3	Double blind	232	3	Diagnosis	0.06
4	Prevalence	202	4	Hypothalamic activation	0.06
5	Chronic cluster headache	146	5	Disability	0.06
6	Pain	134	6	Conjunctival injection	0.06
7	Chronic migraine	126	7	Cervicogenic headache	0.06
8	Deep brain stimulation	118	8	Follow up	0.06
9	Occipital nerve stimulation	117	9	Clinical features	0.06
10	Management	116	10	CGRP	0.06

**Figure 12:**
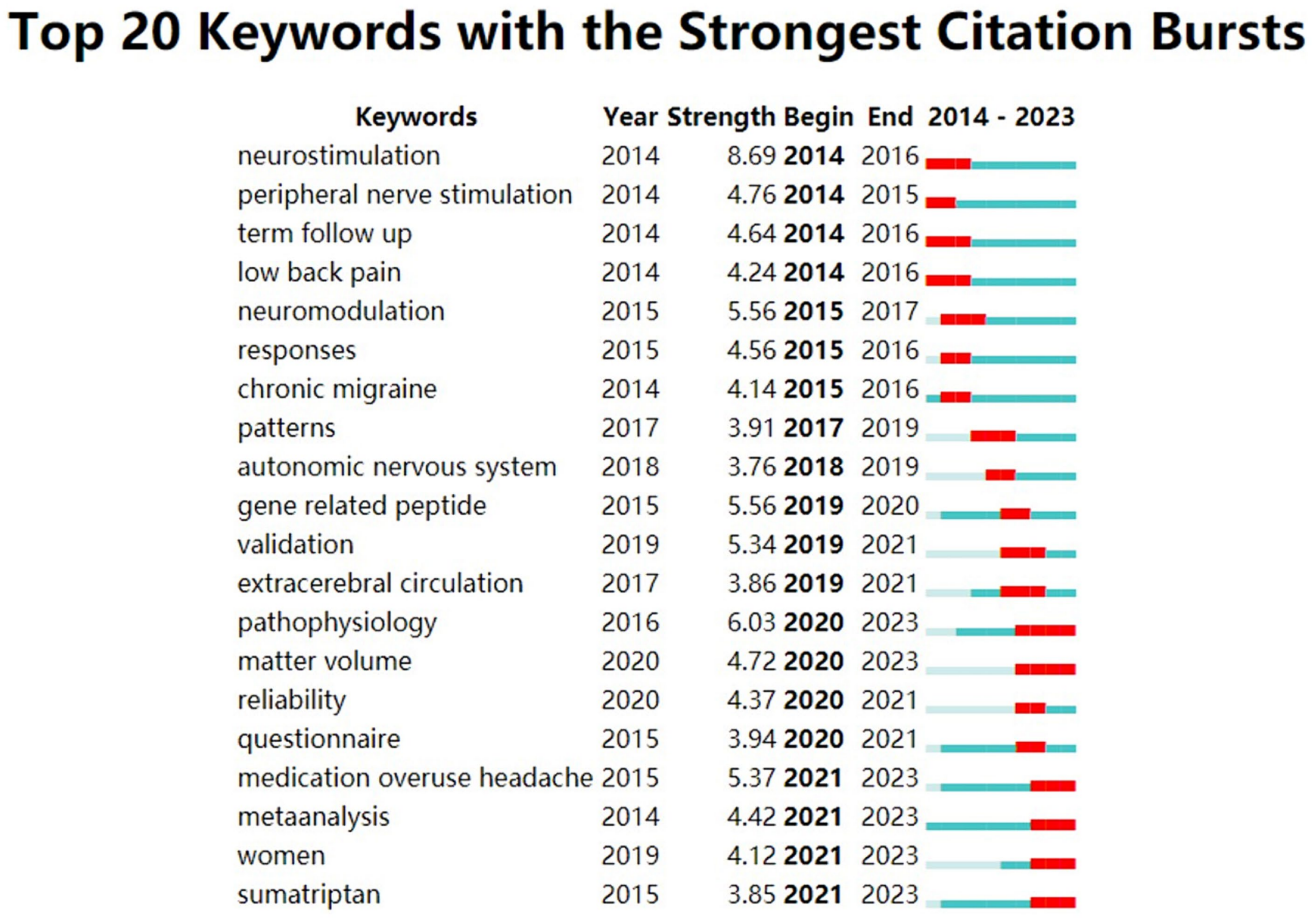
Top 20 keywords with the strongest citation bursts related to cluster headaches (CHs). The begin column demonstrated the start year of the keyword.

**Figure 13:**
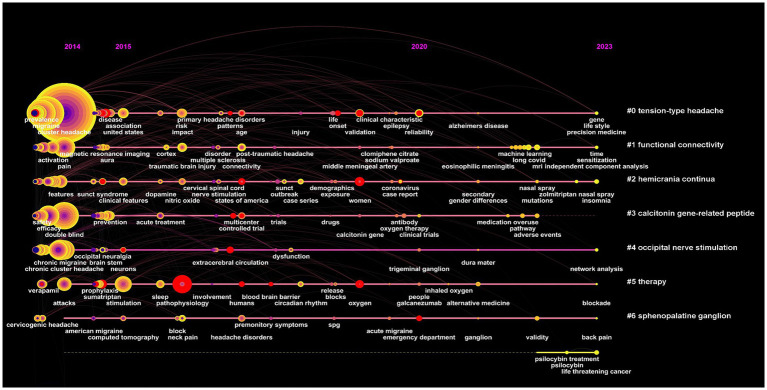
Timeline map of keywords related to cluster headaches (CHs).

Keyword burst refers to a type of words that appear frequently or grows rapidly over a period of time and can be used to predict research trends. The top 20 keywords with the strongest citation explosion from 2014 to 2023 are shown in Figure 12. From the map, we can deduce that from 2014 to 2018, research focused on therapeutic techniques (peripheral nerve stimulation) and diseases (chronic migraine) related to CHs, while from 2019 to 2023, research perspectives were more diverse. Research on CHs should focus more on evidence-based medical (EBM) methodology design in clinical research (questionnaire, follow-up, and meta-analysis). A keyword timeline was generated by taking the cluster to which the node belongs as the vertical axis and the publication time as the horizontal axis. It clearly presents the evolutionary trends of various time periods related to research on CHs (Figure 13). The time span of “cortex” was found to be the longest, and the disease most associated with CHs is insomnia, which appears in 2023.

Calcitonin gene-related peptide (CGRP) is a potent vasodilator expressed by central and peripheral neurons (66). Related studies have shown that CGRP is a major neuronal messenger in CHs, and that there is a clear association between differences in CGRP levels and CH bouts (67). The core of EBM is that medical decisions should be based on objective research results (68). Meta-analyses or the use of the rigorous design of double-blind, questionnaire, and follow-up design randomized controlled trials (RCTs) has a high level of recommendation in EBM, which is conducive to the promotion of scientific decision-making and development of clinical medicine (69, 70).

Occipital nerve stimulation was found to be the most beneficial peripheral nerve stimulation technique in drug-resistant CHs (71). Researchers applied this technique to a retrospective observational trial with consecutive samples; the results showed that patients had a reduced weekly rate of episodes, improved pain intensity, and reduced intake of oral medications. Furthermore, treatment of CHs using occipital nerve stimulation was found to be effective and safe, with no adverse effects (72).

CHs and chronic migraines are closely associated, sharing common clinical features such as unilateral pain sites, similar inducing factors, tretinoin response, and therapeutic effects of neuromodulation (73). CHs and insomnia are often mutually linked. Insomnia is thought to be a possible trigger for CH attacks, and it has also been demonstrated that sleep in patients with CHs is negatively affected during both a CH attack and remission, with major symptoms consistent with insomnia (74).

## 4 Conclusion

This study included 1909 articles on CHs from 2014 to 2023 obtained from the WOSCC database as data materials. By applying bibliometric methods, using CiteSpace, VOSviewer, and Excel software to draw visual knowledge maps, we could clearly present the research status and predict the research hotspots and frontiers of CHs in the past decade. The main finding is that in the past decade, the overall annual publication volume of articles related to CHs has been increasing year by year; therefore, the development prospects are promising. The 1909 articles contained six types of literature, among which the proportion of original research articles was the highest (1,270 articles, 66.53%), published in 201 journals, with *Cephalalgia* showing the highest publication volume (439 articles, 23.00%). Furthermore, the *Lancet* had the highest impact factor (IF = 168.9). The USA was the country with the most published papers (584 articles, 30.60%), University of London was the research institution with the most published papers (142 articles, 7.44%), and Goodsby, Peter J was the most prolific author (38 articles, 1.99%).

The hotspots and frontiers of future research on CHs are suggested to be as follows: in basic medicine, increasing attention should be paid to pathophysiology of CHs, especially increasing research on the pathogenesis mediated by CGRP; in clinical medicine, more attention should be paid to the design of EBM methodology, especially the strict design of double-blind, questionnaire, and follow-up in RCTs, using high-quality articles for meta-analyses, recommending a high level of evidence; therapeutic techniques need to be further explored, suggesting the implementation of transcranial magnetic stimulation of the cortex and stimulation of the sphinopalatine ganglia and occipital nerve to achieve peripheral neuromodulation. Furthermore, chronic migraine and insomnia are inextricably linked to CHs.

In the past decade, research on CHs has been mainly studied in the USA and England, which may be related to each country’s economic development level. Other countries should increase their attention to medical technology development. Research institutions for CHs are dominated by comprehensive universities, which may be related to the academic resources and research atmosphere of the universities. Research institutes or centers for specific disciplines should increase their participation in scientific research on CHs. There are more domestic exchanges and less cross-border exchanges among various research institutions. It was observed that the authors have fixed partners and lack cooperation with other team authors (75, 76). If authors from different research directions break through the constraints of profession, discipline, team, institution, and region, research on CHs will achieve greater progression (77, 78).

## Data availability statement

The datasets presented in this study can be found in online repositories. The names of the repository/repositories and accession number(s) can be found in the article/supplementary material.

## Author contributions

QM: Conceptualization, Methodology, Supervision, Writing – review & editing. SX: Project administration, Writing – original draft. YW: Data curation, Investigation, Writing – review & editing. DW: Data curation, Investigation, Writing – review & editing. GH: Formal analysis, Resources, Writing – review & editing. ZL: Formal analysis, Writing – review & editing. XZ: Software, Visualization, Writing – review & editing. ZC: Funding acquisition, Validation, Writing – review & editing.
